# Reciprocal Negative Regulation Between Lmx1a and Lmo4 Is Required for Inner Ear Formation

**DOI:** 10.1523/JNEUROSCI.2484-17.2018

**Published:** 2018-06-06

**Authors:** Yanhan Huang, Jennifer Hill, Andrew Yatteau, Loksum Wong, Tao Jiang, Jelena Petrovic, Lin Gan, Lijin Dong, Doris K. Wu

**Affiliations:** ^1^National Institute on Deafness and Other Communication Disorders,; ^2^National Eye Institute, National Institutes of Health, Bethesda, Maryland 20892, and; ^3^Department of Neurobiology and Anatomy, University of Rochester, Rochester, New York 14642

**Keywords:** cochlea, cristae, development, genetics, inner ear, semicircular canals

## Abstract

LIM-domain containing transcription factors (LIM-TFs) are conserved factors important for embryogenesis. The specificity of these factors in transcriptional regulation is conferred by the complexes that they form with other proteins such as LIM-domain-binding (Ldb) proteins and LIM-domain only (LMO) proteins. Unlike LIM-TFs, these proteins do not bind DNA directly. LMO proteins are negative regulators of LIM-TFs and function by competing with LIM-TFs for binding to Ldb's. Although the LIM-TF Lmx1a is expressed in the developing mouse hindbrain, which provides many of the extrinsic signals for inner ear formation, conditional knock-out embryos of both sexes show that the inner ear source of Lmx1a is the major contributor of ear patterning. In addition, we have found that the reciprocal interaction between Lmx1a and Lmo4 (a LMO protein within the inner ear) mediates the formation of both vestibular and auditory structures. Lmo4 negatively regulates Lmx1a to form the three sensory cristae, the anterior semicircular canal, and the shape of the utricle in the vestibule. Furthermore, this negative regulation blocks ectopic sensory formation in the cochlea. In contrast, Lmx1a negatively regulates Lmo4 in mediating epithelial resorption of the canal pouch, which gives rise to the anterior and posterior semicircular canals. We also found that Lmx1a is independently required for the formation of the endolymphatic duct and hair cells in the basal cochlear region.

**SIGNIFICANCE STATEMENT** The mammalian inner ear is a structurally complex organ responsible for detecting sound and maintaining balance. Failure to form the intricate 3D structure of this organ properly during development most likely will result in sensory deficits on some level. Here, we provide genetic evidence that a transcription factor, Lmx1a, interacts with its negative regulator, Lmo4, to pattern various vestibular and auditory components of the mammalian inner ear. Identifying these key molecules that mediate formation of this important sensory organ will be helpful for designing strategies and therapeutics to alleviate hearing loss and balance disorders.

## Introduction

The ability to detect sound and maintain balance is mediated by the auditory and vestibular components of the intricate inner ear. In mammals, the auditory component is the snail-shaped cochlea. The vestibular component consists of sensory maculae of the utricle and saccule that detect linear acceleration. In addition, the three semicircular canals and their associated ampullae that house the sensory cristae are responsible for detecting angular acceleration. Proper morphogenesis of this complex, fluid-filled organ requires a dynamic interplay of intrinsic and extrinsic signaling pathways (Groves and Fekete, 2012; Wu and Kelley, 2012). The hindbrain is a key tissue for providing many of the extrinsic signals required by the ear and some of these signals function indirectly because they are transcription factors; for example, Mafb (Choo et al., 2006; Groves and Fekete, 2012; Wu and Kelley, 2012). In addition, many of these extrinsic molecules are expressed within the inner ear as well during development. For example, the LIM-TF Lmx1a is expressed in both the developing hindbrain and inner ear. The *dreher* mutants are functional nulls for the Lmx1a protein and show developmental defects in both the hindbrain (missing the roof plate) and the inner ear, which is rudimentary in shape and missing the endolymphatic duct (Millonig et al., 2000; Nichols et al., 2008; Koo et al., 2009). Parsing out the extrinsic and intrinsic requirements of Lmx1a or other signaling molecules for the intricate inner ear is important.

Lmx1a belongs to the family of LIM-domain containing transcription factors (LIM-TFs), which are named after three factors Lin11, Isl1, and Mec3, that exhibit similar cysteine and histidine rich zinc fingers in the protein-interacting LIM domain (Rétaux and Bachy, 2002). Studies of *apterous*, a *Drosophila* homolog of *Lmx1a*, have provided insights into the molecular mechanisms of this class of TFs (Cohen et al., 1992; Milán et al., 1998; Milán and Cohen, 1999). LIM-TFs mediate context-specific transcription by forming a complex with cofactors such as Ldbs, also known as CLIM2/NL1/Chip. Transcriptional activities of LIM-TFs are negatively regulated by nuclear LMOs, which compete with LIM-TFs to bind to Ldbs (for review see Rétaux and Bachy, 2002; Gill, 2003). Currently, 12 LIM-TFs and four LMO factors are known in mammals. These 16 factors are implicated in a number of developmental events such as hematopoiesis, limb formation, and neurogenesis, even though their interacting partners are not always clear (Chen et al., 1998; Yamada et al., 1998; Lee et al., 2008). All four known Lmo nuclear proteins are expressed in the developing inner ear (Deng et al., 2006), but only Lmo4 has been shown to be required for both vestibular and cochlear development (Deng et al., 2010; Deng et al., 2014). In *Lmo4* knock-outs, all three semicircular canals plus anterior and posterior ampullae are reported to be missing. The cochlear duct is slightly shortened with a duplicated organ of Corti (Deng et al., 2014). The underlying mechanism of Lmo4 function and its possible relationship with LIM-TF in the inner ear, however, are not known.

In this study, we first investigated the significance of the inner ear source of *Lmx1a* on ear development by generating an inner-ear-specific conditional knock-out (CKO) of *Lmx1a*. Phenotypes of *Lmx1a* CKO recapitulate those of the *dreher* mutants, suggesting that intrinsic *Lmx1a* is a key mediator of inner ear development. Furthermore, we provide genetic evidence that Lmx1a and Lmo4 negatively regulate each other to form various structures of the inner ear.

## Materials and Methods

### 

#### 

##### Generation of *Lmx1a^lox^* mice.

**Figure 1.**
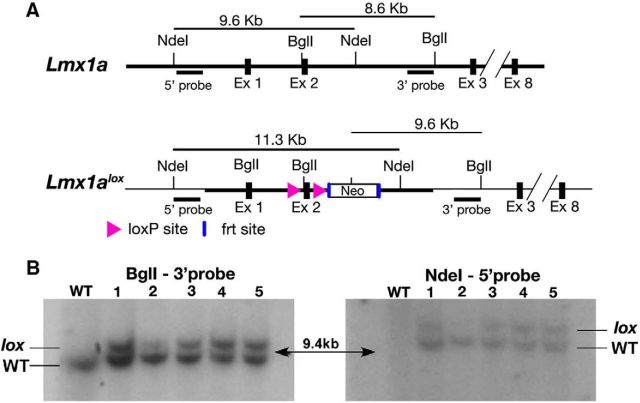
*Lmx1a* conditional mouse knock-out scheme. ***A***, Genomic organization of mouse *Lmx1a* gene and *Lmx1a*^lox^ alleles are shown. *LoxP* sites were positioned to flank exon 2 of the target gene using the ET recombination technique. Homologous recombination events in R1 ES cells were selected by genomic Southern analysis. ***B***, Using 5′ and 3′ DNA probes against genomic DNA from wild-type (WT) and various ES clones (1–5) digested with NdeI and BglI, respectively.

The *Lmx1a*^lox^ allele was generated by homologous recombination in mouse embryonic stem (ES) cells as described previously (Thomas and Capecchi, 1987). A targeting vector of replacement type was constructed first by Red/ET (RecE and RecT) recombination (Testa et al., 2003) using a bacterial artificial chromosome clone encompassing the entire locus of the target gene (Source BioScience). The targeting vector had two *LoxP* sites flanking a 491 bp exon 2 of *Lmx1a*. The mouse R1 ES cells derived from SV129 background (Nagy et al., 1993) were electroporated with the linearized targeting vector and homologous recombination events were selected by Southern blotting with appropriate probes (see Fig. 1) as described previously (Thomas and Capecchi, 1987). Correctly targeted ES clones were introduced into recipient blastocyst embryos by microinjection and the two high percentage male chimeras generated were crossed with wild-type C57bl/6 females to recover germline-transmitted founders carrying the *lox* allele. One of the founders was bred to a ubiquitous *flp* strain (JAX #005703, RRID:IMSR_JAX:005703) to remove the neomycin cassette. The targeted *Lmx1a* locus of *Lmx1a*^lox/lox^ mice after *flp* recombination was further verified with genomic PCR.

##### Mouse strains.

To generate *Lmx1a* conditional mutants, *Foxg1*^Cre^ or *Sox9-IRES-Cre* (abbreviated as *Sox9*^cre^) was first bred to heterozygotes of *dreher*, *Lmx1a*^dr/+^, to generate double heterozygotes. Double heterozygotes males were bred to *Lmx1a*^lox/lox^ females and embryos were harvested at various ages as indicated. *Lmo4*^−/−^, *Lmx1a*^dr/dr^, and compound mutants of *Lmx1a* and *Lmo4* were generated by crossing *Lmx1a*^dr/+^*;Lmo4*^+/−^ double heterozygotes (Hébert and McConnell, 2000; Akiyama et al., 2005; Chizhikov et al., 2006; Deng et al., 2010). All animal studies were conducted under the approved animal protocol (#1212–17) and according to the National Institutes of Health's animal user guidelines.

##### Paint fill, immunohistochemistry, *in situ* hybridization, and imaging.

Paint-fill and *in situ* hybridization of mouse specimens were performed as described previously (Morsli et al., 1998). For whole-mount immunostaining, dissected inner ears were incubated with blocking solution (4% donkey serum + 0.2% Triton X-100 in 1× PBS) for 1 h before adding primary antibodies and incubating overnight at 4°C with gentle rocking. After washing with 1× PBS, specimens were incubated with secondary antibodies diluted in blocking solution for 2 h and then specimens were washed and mounted on microscope slides with Prolong Gold antifade. Similar procedures were performed for immunostaining on sections except PBS containing 0.3% Triton X-100 (PBX) was used as the preblock solution. Samples subjected to immunohistochemistry were imaged using a Zeiss LSM780. Specimens processed for *in situ* hybridization were imaged using a Zeiss AxioPlan2 Microscope System.

Primary antibodies and dilutions used for the studies are listed as follow: rabbit anti-Myosin VIIa (1:250, Proteus Biosciences, 25-6790, RRID:AB_2314840), goat anti-P75Ngfr (1:250, Neuromics, GT15057-100, RRID:AB_1611758), mouse anti-2H3 (1:1000, Developmental Studies Hybridoma Bank, RRID:AB_2314897), goat anti-Jagged 1 (anti-Jag1, 1:200, Santa Cruz Biotechnology, SC-6011, RRID:AB_649689), goat anti-Sox2 (1:400, Santa Cruz Biotechnology, SC-17320, RRID:AB_2286684), mouse anti-GFP (1:100, Invitrogen, A11120, RRID:AB_221568), and rabbit anti-Lmx1a (1:500, Millipore, AB10533, RRID:AB_10805970). Secondary antibodies were obtained from Invitrogen and used at 1:250 dilution: Alexa Fluor 350 goat anti-rabbit IgG (H+L) (RRID:AB_2534101), Alexa Fluor 488 donkey anti-rabbit IgG (H+L) (RRID:AB_141708), Alexa Fluor 488 and 555 donkey anti-goat IgG (H+L) (RRID:AB_142672 and RRID:AB_141788), and Alexa Fluor 647 donkey anti-mouse IgG (H+L) (RRID:AB_162542). FITC or rhodamine-labeled phalloidin (1:125, Life Technologies, F432 and R415, RRID:AB_2572408) was used.

##### Hindbrain analyses.

Embryos for hindbrain analyses were examined under a dissecting microscope at the time of harvest at embryonic day 10.5 (E10.5) and the size of the roof plate was recorded as either normal or reduced/abnormal. After genotyping, the size of the roof plate was compared with various genotypes. Because the size of roof plate also varies in normal embryos at this age, smaller roof plates were found in both wild-type and mutant embryos (see Results).

##### Electroporation and analyses.

Expression plasmids of *pMES-GFP* or *pMES-Lmx1b-GFP* (4 μg/μl) were electroporated into the presumptive anterior crista region of the right otocyst at E3.5, equivalent to a Hamburger–Hamilton (HH) staging of stage 22–23 (Hamburger and Hamilton, 1951), *in ovo* as described previously (Chang et al., 2008). Twenty-four hours after electroporation (equivalent to HH25-26), embryos were harvested. The efficiency of the electroporation was assessed by the GFP coverage within the anterior crista region and only the specimens with satisfactory GFP expression were cryosectioned into sets of three and processed for *Bmp4 in situ* hybridization and anti-Sox2 and anti-Jag1 immunostaining. For each specimen, qualitative assessment of differential gene expression pattern was made by comparing the electroporated right ear with the nonelectroporated left ear as an internal control. The assessment of gene expression changes was conducted separately by two individuals who were blinded to the plasmids used in the electroporation.

##### Experimental design and statistical analysis.

Both male and female mice were used in this study and they were maintained on a mixed C3H3/C57BL6 background. Numbers of samples used for each experiment are listed in the Results. Statistical analyses were performed using Student's *t* test or χ^2^ test and all *p*-values are reported with each test.

## Results

### Inner ear source of *Lmx1a* is required for inner ear patterning

**Figure 2.**
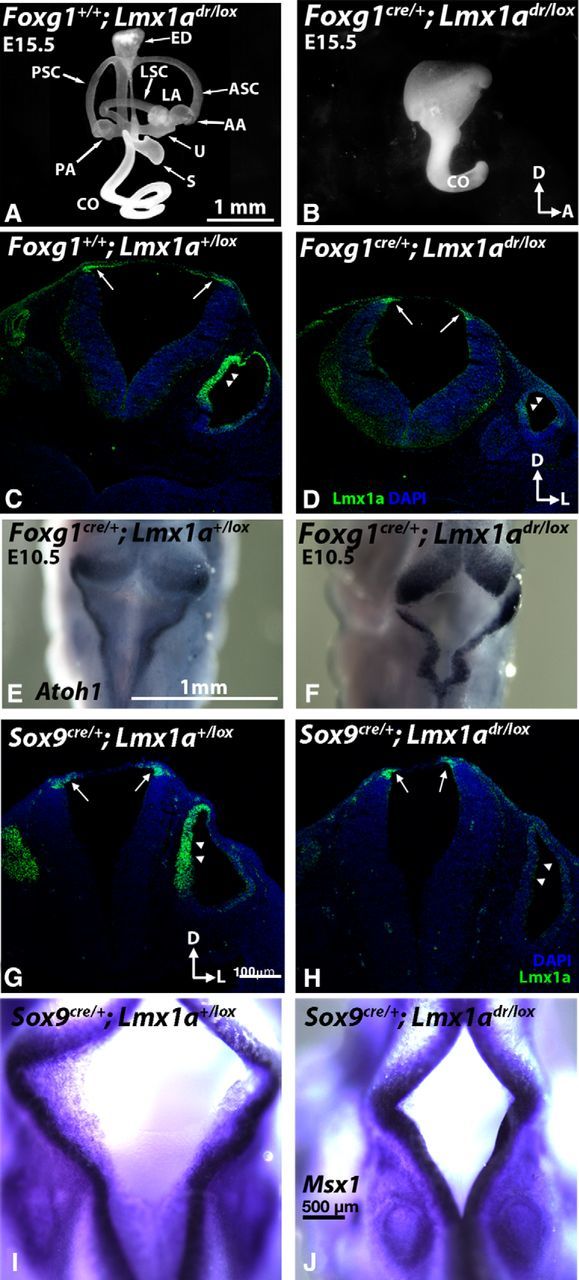
Dysmorphic inner ears have normal Lmx1a immunoreactivities in the hindbrain of *Foxg1*^cre/+^*;Lmx1a*^dr/lox^ and *Sox9*^cre/+^*;Lmx1a*^dr/lox^ CKO mutants. ***A***, ***B***, Paint-filled inner ears of control *Foxg1*^+/+^*;Lmx1a*^dr/lox^ (***A***, *n* = 4) and *Foxg1*^cre/+^*;Lmx1a*^dr/lox^ CKO (***B***, *n* = 8) embryos at E15.5. The dysmorphic inner ear in the conditional mutant is indistinguishable from that of the *dreher*. ***C***–***F***, Lmx1a immunostaining (***C***, ***D***) and *Atoh1* expression (***E***, ***F***) in the hindbrain of *Foxg1*^+/+^*;Lmx1a*^+/lox^ control (***C***, ***E***) and *Foxg1*^cre/+^*;Lmx1a*^dr/lox^ CKO (***D***, ***F***) embryos at E10.5. ***C***, ***D***, Lmx1a immunostaining is markedly reduced in the otic epithelium (arrowheads) but maintained in the hindbrain (arrows) of *Foxg1*^cre/+^*;Lmx1a*^dr/lox^ CKO. No downregulation of *Atoh1* expression is observed in the rhombic lip of *Foxg1*^cre/+^*;Lmx1a*^dr/lox^ CKO hindbrains (***F***, *n* = 3) compared with *Foxg1*^cre/+^*;Lmx1a*^+/lox^ controls. ***G***–***I***, Inner ears and hindbrain phenotypes of *Sox9*^cre/+^*;Lmx1a*^dr/lox^ CKO mutants. ***G***, ***H***, Anti-Lmx1a staining in the *Sox9*^cre/+^*;Lmx1a*^dr/lox^ CKO mutants (***H***) is comparable to the *Sox9*^cre/+^*;Lmx1a*^+/lox^ controls (***G***) in the dorsal hindbrain (arrows), but is much reduced in the inner ear (arrowheads). ***I***, ***J***, *Msx1* expression in the rhombic lip of a control (***I***) and *Sox9*^cre/+^*;Lmx1a*^dr/lox^ CKO mutants (***J***). The diamond-shaped roof plate is slightly smaller in the CKO mutants. AA, Anterior ampulla; ASC, anterior semicircular canal; CO, cochlear duct; ED, endolymphatic duct; LA, lateral ampulla; LSC, lateral semicircular canal, PA, posterior ampulla; PSC, posterior semicircular canal; S, saccule; U, utricle. Orientations: A, anterior; D, dorsal; L, lateral. Anterior is toward the top of the panel for ***E***, ***F***, ***I***, and ***J***.

To investigate the importance of the inner ear source of *Lmx1a* on ear development, we generated *Lmx1a*^lox/lox^ mice (Fig. 1). These mice are viable and breed well with no apparent phenotype. Inner-ear-specific *Lmx1a* knock-out was generated by using *Foxg1*^cre^, which expresses cre recombinase in the otic epithelium starting at the otic placode stage, but does not express cre in the roof plate or rhombic lip of the hindbrain (Hébert and McConnell, 2000). Inner ears of *Foxg1*^cre/+^*;Lmx1a*^dr/lox^ at E15.5 display a rudimentary phenotype that is indistinguishable from the *dreher* mutants: absence of the endolymphatic duct and three semicircular canals, as well as a shortened cochlear duct (Fig. 2*A*,*B*; *n* = 8; Nichols et al., 2008; Koo et al., 2009). Compared with controls (Fig. 2*C*), Lmx1a immunoreactivity is present in the dorsal hindbrain (Fig. 2*D*, arrows), but is much reduced in the otic epithelium of *Foxg1*^cre/+^*;Lmx1a*^dr/lox^ CKO at E10.5 (Fig. 2*D*, arrowheads). The residual immunostaining detected in the otic epithelium is attributed to the presence of the functionally null Lmx1a protein encoded by the *dreher* allele (Millonig et al., 2000). These expression results suggest that Lmx1a functions in these CKO are absent in the inner ear but intact in the hindbrain. Consistently, expression of *Atoh1* in the rhombic lip at the edge of the roof plate was found in the *Foxg1*^cre/+^*;Lmx1a*^dr/lox^ embryos (Fig. 2*E*,*F*), though much reduced in the *dreher* mutants (Millonig et al., 2000). However, the size of roof plates in *Foxg1*^cre/+^*;Lmx1a*^dr/lox^ embryos was invariably smaller or abnormal compared with controls (*n* = 10 from five litters). The cause for this hindbrain phenotype is not clear because cre is not expressed in the rhombic lip or roof plate of *Foxg1*^cre/+^*;Lmx1a*^dr/lox^ embryos (Hébert and McConnell, 2000).

Although Lmx1a expression in the dorsal hindbrain is not disrupted in the *Foxg1*^cre/+^*;Lmx1a*^dr/lox^ embryos, it is possible that this defect in the hindbrain affected ear formation. To address the possible impact of roof plate abnormality on inner ear development, we generated another inner-ear-specific *Sox9*^cre/+^*;Lmx1a*^dr/lox^ using *Sox9*^cre^, in which cre reporter activity is present in the inner ear and its adjacent mesenchyme but not in the hindbrain (Raft et al., 2014). As expected, the *Sox9*^cre/+^*;Lmx1a*^dr/lox^ CKO ears showed a strong reduction of Lmx1a immunostaining in the otic epithelium but not the hindbrain, similar to that of the *Foxg1*^cre/+^*;Lmx1a*^dr/lox^ CKO (Fig. 2*G*, *H*). The paint-filled inner ears of *Sox9*^cre/+^*;Lmx1a*^dr/lox^ CKO were also indistinguishable from those of the *Foxg1*^cre/+^*;Lmx1a*^dr/lox^ CKO or *dreher* mutants (*n* = 8, data not shown). Despite the fully penetrant inner ear phenotypes in *Sox9*^cre/+^*;Lmx1a*^dr/lox^ CKO (*n* = 8/8), the roof plate phenotype was much milder than those in *Foxg1*^cre/+^*;Lmx1a*^dr/lox^ CKO (Fig. 2*I*,*J*) and some hindbrains in *Sox9*^cre/+^*;Lmx1a*^dr/lox^ CKO were indistinguishable from controls at the time of harvest (Fig. 2*H*, *n* = 4/7 from 5 litters). Therefore, the inner ear and roof plate phenotypes are not well correlated in the *Sox9*^cre/+^*;Lmx1a*^dr/lox^ CKO strain. Based on the results from these two cre strains, we attributed the inner ear source of *Lmx1a* to play a more important role in mediating inner ear formation than *Lmx1a* expressed in the hindbrain.

### *Foxg1*^cre/+^*;Lmx1a*^dr/lox^ CKO mutants have similar sensory defects as *dreher*

**Figure 3.**
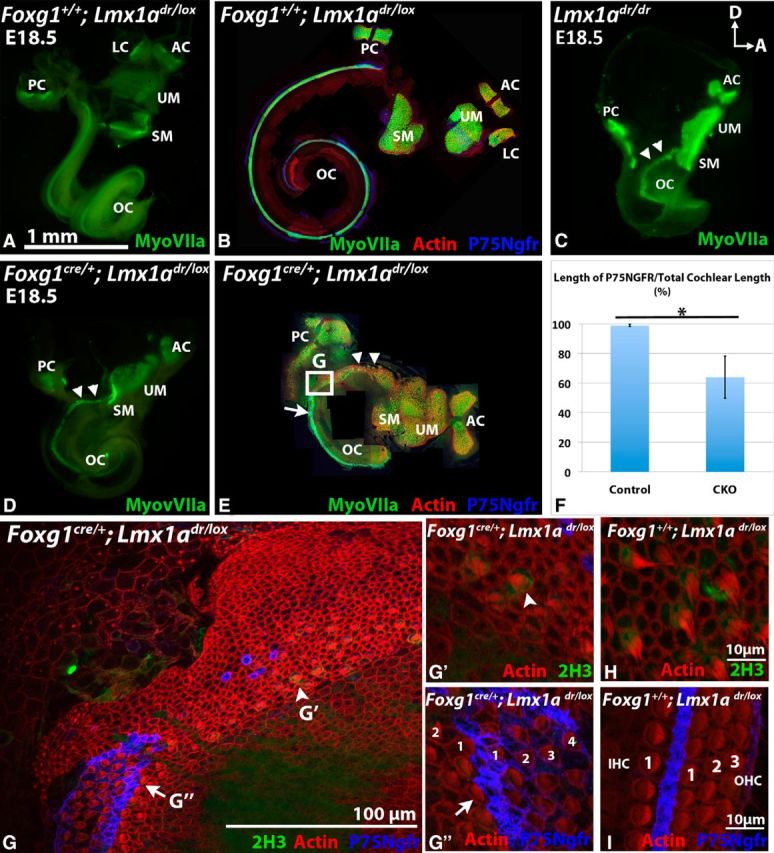
Sensory defects in *Foxg1*^cre/+^*;Lmx1a*^dr/lox^ CKO mutants. ***A***–***E***, Hair cells labeled with anti-myosin VIIa antibodies are in green, actin labeled with rhodamine-phalloidin are in red, and pillar cells labeled with anti-P75Ngfr are in blue. ***G***–***I***, Developing calyxes labeled with anti-neurofilament antibodies (2H3) are in green. ***A***, ***B***, Whole-mount (***A***) and dissected (***B***) inner ears of *Foxg1*^+/+^*;Lmx1a*^dr/lox^ controls. Vestibular and auditory sensory organs are compartmentalized in chambers (***A***; *n* = 9). ***C***–***E***, Whole mount of *dreher* (***C***; *n* = 2) and whole (***D***; *n* = 5) and flat (***E***; *n* = 5) mounts of *Foxg1*^cre/+^*;Lmx1a*^dr/lox^ CKO ears showing individual sensory organs are distinguishable but often fused with each other. The double arrowheads indicate the saccular macula and the organ of Corti are continuous with each other. In addition to the lack of a distinct lateral crista, the posterior crista is malformed (***D***, ***E***). Sensory organs are identified based on their locations in the inner ear. ***D*** and ***E*** are composites of images taken at a higher magnification. ***F***, Percentages of the length of P75Ngfr-positive cochlear region of *Foxg1*^cre/+^*;Lmx1a*^dr/lox^ CKO (*n* = 5) relative to that of *Foxg1*^+/+^*;Lmx1a*^dr/lox^ and *Foxg1*^cre/+^*;Lmx1a*^lox/+^ controls (*n* = 8, *p* < 0.005 (*p* = 0.0012, Student's *t* test)). ***G***, Higher magnification of the square region in ***E***. Continuous P75Ngfr expression only starts at the midbase (***G′′***) and not at the base (***G′***) of the cochlea. At the base of the CKO cochlea, 2H3-positive staining surrounding the hair cells is observed (***G′***; *n* = 3), which resembles developing calyxes in the maculae of controls (***H***; *n* = 4). Multiple rows of hair cells are present in the midbase of mutants (***G′′***) compared with the one row of inner hair cells (IHC) and three rows of outer hair cells (OHC) in *Foxg1*^+/+^*;Lmx1a*^dr/lox^ controls taken from a comparable basal cochlear region (***I***). AC, Anterior crista; OC, organ of Corti; LC, lateral crista; PC, posterior crista; SM, saccular macula; UM, utricular macula. For orientations, please refer to Figure 2.

Next, we investigated the sensory organs within the *Foxg1*^cre/+^*;Lmx1a*^dr/lox^ CKO ears using anti-myosin VIIa antibodies and phalloidin, which label sensory hair cells and their stereociliary bundles, respectively (Fig. 3). In *Foxg1*^+/+^*;Lmx1a*^dr/lox^ controls, myosin VIIa-positive sensory epithelia were compartmentalized into distinct chambers (Fig. 3*A*,*B*). In contrast, most of the sensory organs within the *Foxg1*^cre/+^*;Lmx1a*^dr/lox^ CKO ears were distinguishable but the maculae of the utricle and saccule and the organ of Corti were continuous and not well separated from each other (Fig. 3*D*,*E*, UM, SM, OC, arrowheads), similar to the *dreher* mutants (Fig. 3*C*, arrowheads; Nichols et al., 2008; Koo et al., 2009).

Another salient feature described for the *dreher* mutants is the presence of vestibular-like hair cells in the basal region of the cochlea (Nichols et al., 2008; Koo et al., 2009). Similar features are present in the *Foxg1*^cre/+^*;Lmx1a*^dr/lox^ CKO mutants. P75 Ngfr immunostaining, which is associated with the pillar cells of the organ of Corti, was missing from the basal cochlea of the conditional mutants (Fig. 3*F*,*G*,*G′*). Some of the hair cells displayed short stereocilia and the cell bodies were surrounded by anti-neurofilament antibody staining that resembled developing calyxes in the maculae (Fig. 3*G*,*G′*,*H*; Koo et al., 2009). Beyond this basal region, the organ of Corti was disorganized with multiple rows of inner and outer hair cells (Fig. 3*G*,*G′′*) compared with controls (Fig. 3*I*). Based on the paint-fill results and the sensory organ analyses, the inner ears of *Foxg1*^cre/+^*;Lmx1a*^dr/lox^ CKO embryos recapitulate well the phenotypes described previously for the *dreher* mutants (Nichols et al., 2008; Koo et al., 2009).

### Genetic interactions between *Lmx1a* and *Lmo4* in inner ear patterning

We next addressed the question of how Lmx1a functions are regulated in the inner ear. A large number of studies in both vertebrates and invertebrates suggest that LIM-TFs are negatively regulated by LMO proteins during embryogenesis (for review see, Bach, 2000; Sang et al., 2014). *Lmo4* knock-out inner ears in the C57BL6 background were reported to lack all three semicircular canals and the anterior and posterior ampullae. However, the endolymphatic duct, lateral ampulla, utricle, saccule, and cochlea are present (Deng et al., 2010, 2014). Although it was not readily apparent how Lmo4 and Lmx1a might interact with each other based on their mutant phenotypes alone, we investigated a possible interaction between these two proteins using a genetic approach by generating compound mutants of *Lmx1a* and *Lmo4*.

**Figure 4.**
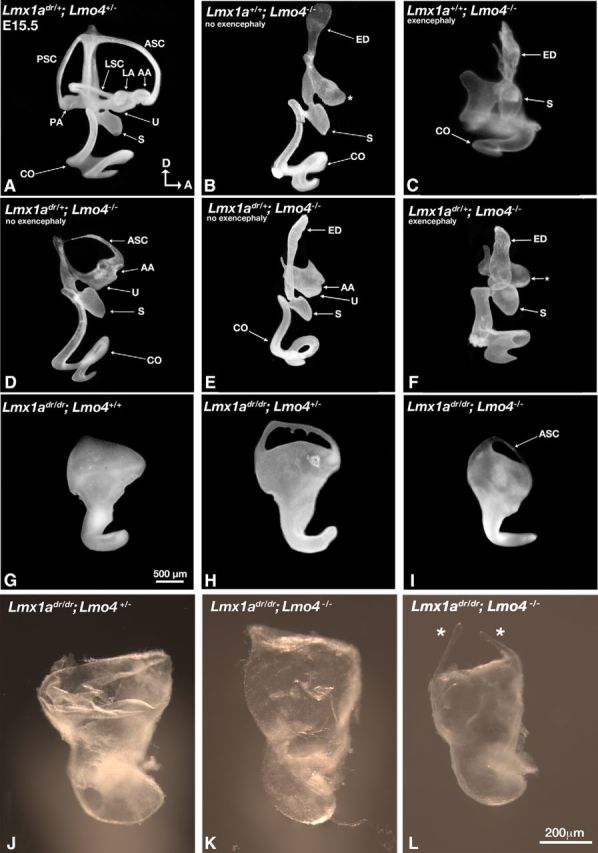
Paint-filled and dissected inner ears of *Lmx1a* and *Lmo4* compound mutants. ***A***, *Lmx1a*^dr/+^*;Lmo4*^+/−^ inner ears are normal (*n* = 4). ***B***, *Lmx1a*^+/+^*;Lmo4*^−/−^ inner ear in an embryo without exencephaly lacking semicircular canals, ampullae, and a well defined utricle (asterisk, *n* = 4/8). ***C***, *Lmx1a*^+/+^*;Lmo4*^−/−^ inner ear in an embryo with exencephaly showing the presence of a saccule, a distorted cochlear duct and the absence of canals (***C***, *n* = 8/9). ***D***–***F***, Most *Lmx1a*^dr/+^*;Lmo4*^−/−^ inner ears in embryos without exencephaly consist of an anterior ampulla (AA), an anterior canal (asc), and a well demarcated utricle (***D***, *n* = 8/11), but some only show an ampulla without a canal (***E***, *n* = 2/11). In contrast, a well defined utricle (asterisk), ampulla, and canal are not recovered in *Lmx1a*^dr/+^*;Lmo4*^−/−^ ears in embryos with exencephaly (***F***, *n* = 5/8). The other three specimens have no utricle. Some *Lmx1a*^dr/dr^*;Lmo4*^+/−^ (***H***, *n* = 7/11) and *Lmx1a*^dr/dr^*;Lmo4*^−/−^ (***I***, *n* = 1/2, L, *n* = 1) show the presence of a canal compared with the *dreher* mutants (***G***, *n* = 4). Other *Lmx1a*^dr/dr^*;Lmo4*^+/−^ (***J***, *n* = 2) and *Lmx1a*^dr/dr^*;Lmo4*^−/−^ (***K***, *n* = 1) dissected ears resemble the *dreher*. ***J***–***L***, Dissected membranous labyrinths of *Lmx1a*^dr/dr^*;Lmo4*^+/−^ (***J***, *n* = 2) and *Lmx1a*^dr/dr^*;Lmo4*^−/−^ specimens (***K***, ***L***). Asterisks in ***L*** show the presence of a canal. ASC, Anterior semicircular canal; LSC, lateral semicircular canal; PSC, posterior semicircular canal. For other abbreviations, please refer to Figure 2.

Although none of the *dreher* or *Lmx1a* conditional mutants exhibited exencephaly (open brain phenotype), ∼50% of our *Lmo4* knock-outs in the mixed C3H3/C57BL6 background showed exencephaly, which has been described previously (Table 1; Lee et al., 2005; Deng et al., 2010). However, inner ear defects found among specimens with or without exencephaly are largely similar except the cochlear duct is distorted in embryos with exencephaly (Fig. 4*B*,*C*, Table 1; Deng et al., 2010). Nevertheless, the phenotypes in the vestibule were more severe than those reported previously (Deng et al., 2010). For example, all three ampullae and their associated semicircular canals were usually missing and the utricle was often not well demarcated by paint fill (Fig. 4*B*,*C*, asterisk, Table 1, ampullae and canals, *n* = 15/17; utricle, *n* = 9/17), compared with controls and *Lmo4* knock-outs in the C57BL6 background (Fig. 4*A*; Deng et al., 2010). However, the presence of the endolymphatic duct, saccule, and cochlear duct were similar to the *Lmo4* knock-out in C57BL6 background. These phenotypic differences between the two *Lmo4* knock-out strains are most likely due to their genetic background.

**Table 1.** Morphological analyses of inner ears in *Lmx1a* and *Lmo4* genotypes

Genotypes	ED	Ampulla	Canal	Utricle	Saccule	Cochlea
*Lmo4*^−/−^ (exencephaly)	9/9	1/9^+^	1/9^+^	4/9	9/9	9/9^++^
*Lmo4*^−/−^ (no exencephaly)	8/8	1/8^a^	0/8^b^	4/8	7/8	4/8
			1/8^+^			1/8^++^
*Lmx1a*^dr/+^*;Lmo4*^−/−^	8/8	1/8	1/8	5/8	8/8	2/8
(exencephaly)		1/8^+^	1/8^+^			6/8^++^
*Lmx1a*^dr/+^*;Lmo4*^−/−^	11/11	10/11^a^	8/11^b^	11/11	11/11	11/11
(no exencephaly)		1/11^+^	1/11^+^			
*Lmx1a*^dr/dr^	0/4	0/4	0/4^c^	0/4	0/4	4/4^++^
*Lmx1a*^dr/dr^*;Lmo4*^+/−^	0/11	0/11	7/11^c^	0/11	0/11	11/11^++^
*Lmx1a*^dr/dr^*;Lmo4*^−/−^	0/4	0/4	2/4	0/4	0/4	4/4^+/+^

+, Lateral ampulla or canal (no denotation: refer to the anterior ampulla or canal); ++, malformed cochlea (refer to Fig. 4*B*,*C* for *Lmo4*^−/−^ without and with exencephaly, respectively; Fig. 4*G* for *Lmx1a*^dr/dr^; and Fig. 4*H*,*I* for *Lmx1a*^dr/dr^*;Lmo4*^+/−^ and *Lmx1a*^dr/dr^*;Lmo4*^−/−^ cochlear images, respectively; no denotation: refer to normal cochlea).

^a^*p* = 0.0006, χ^2^ analysis.

^b^*p* = 0.0015, χ^2^ analysis.

^c^*p* = 0.0289, χ^2^ analysis.

In compound *Lmx1a*^dr/+^*;Lmo4*^−/−^ mutants, the frequency of exencephaly was similar to *Lmo4* knock-outs alone (Table 1). However, some of the ear phenotypes observed in *Lmo4* mutants were rescued in these compound mutants. For example, 10 of 11 *Lmx1a*^dr/+^*;Lmo4*^−/−^ inner ears in embryos without exencephaly showed an anterior ampulla and eight of them showed an accompanying anterior canal (Fig. 4*D*). Figure 4*E* shows an ampulla without a canal (Table 1). Two of 11 specimens also showed a lateral ampulla or a partial lateral canal but the posterior crista and canal were invariably missing (Table 1). All specimens showed a well defined utricle and a normal cochlea and saccule (Fig. 4*D*,*E*, Table 1). These rescued ear structures were less prevalent in the compound mutants with exencephaly (Fig. 4*F*); the utricle was not well defined (Fig. 4*F*, asterisk, *n* = 5/8) and only two of eight specimens possessed an ampulla and canal (data not shown). These results suggest that, although the inner ear phenotypes in *Lmo4* knock-outs with exencephaly are not more severe than those without exencephaly, the rescue of inner ear defects by removing one allele of *Lmx1a* is less efficient in embryos with exencephaly.

Given the postulated stoichiometric relationship between Lmx1a and Lmo4, we also investigated the phenotypes of *dreher* inner ears that lack one allele of *Lmo4*. None of these specimens exhibited exencephaly. Two dissected membranous labyrinths (Fig. 4*J*) and two of seven *Lmx1a*^dr/dr^*;Lmo4*^+/−^ embryos in which both ears were injected with paint (data not shown) showed the *dreher* phenotype (Fig. 4*G*), whereas the remaining five specimens all showed the presence of a canal in one of the two ears (Fig. 4*H*). Four specimens with only one of the two ears injected showed two ears with canals and two without (Table 1). The prevalence of canal recovery in *Lmx1a*^dr/dr^*;Lmo4*^+/−^ ears (Table 1, *n* = 7/11) suggests that Lmx1a also negatively regulates Lmo4 in canal formation, although there is variability in this regulation even between ears within the same genetic background.

In two of the four double-null mutants (*Lmx1a*^dr/dr^*;Lmo4*^−/−^) processed for paint fill or membranous dissection, there was a recovery of an canal (Fig. 4*I*,*L*), similar to *Lmx1a* mutants that lack only one allele of *Lmo4* (Fig. 4*H*, Table 1), and the other two double mutants showed the *dreher* phenotype (Fig. 4*K*). The resemblance of the double mutant phenotype to those of *dreher* suggests that *Lmx1a* is epistatic to *Lmo4* in inner ear development.

### Negative regulation of *Lmx1a* by *Lmo4* is important for crista formation

**Figure 5.**
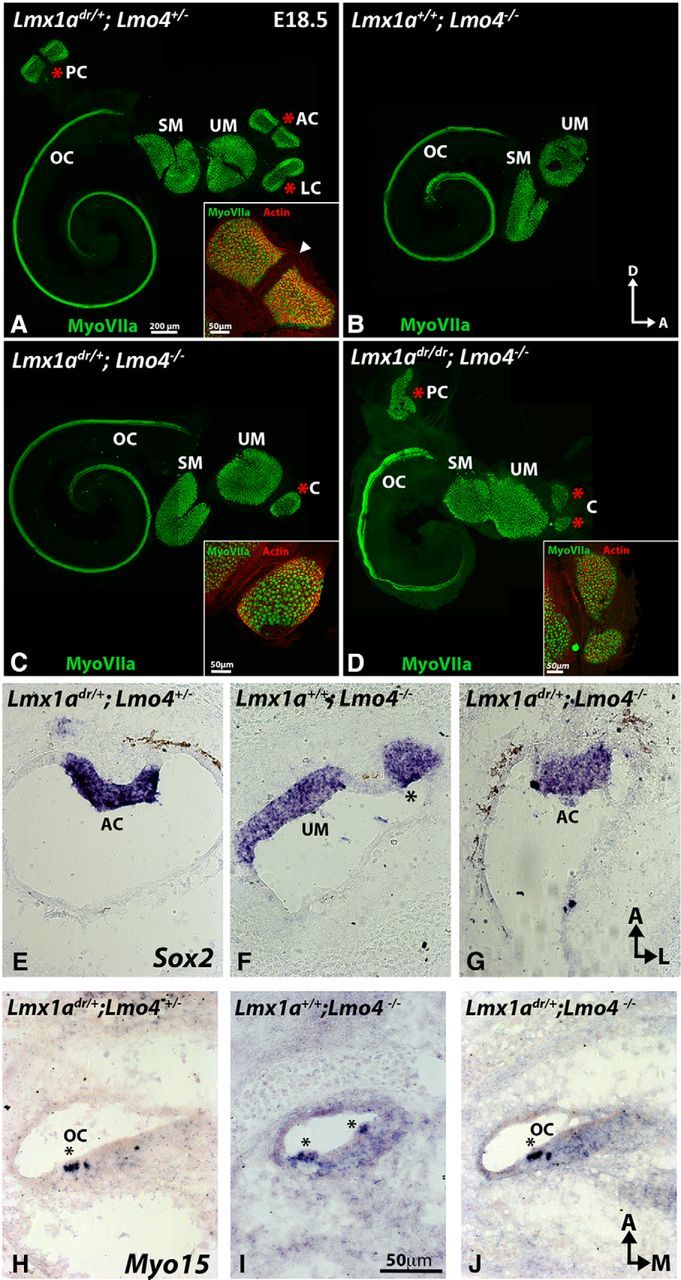
Crista and ectopic organ of Corti formation are inversely related to the presence of *Lmx1a*. ***A***–***D***, Anti-myosin VIIa staining is shown in green, actin staining with rhodamine-phalloidin in red, and cristae are marked with red asterisks. Insets show the anterior crista of control and the anteriorly located crista in mutants. ***A***, *Lmx1a*^dr/+^*;Lmo4*^+/−^ inner ears are normal showing six myosin VIIa-positive sensory organs (*n* = 4). Cruciatum divides the anterior and posterior crista into two equal halves (arrowhead in inset). ***B***, *Lmx1a*^+/+^*;Lmo4*^−/−^ inner ears lack all three sensory cristae (*n* = 2/3), whereas a crista-like sensory organ is present in *Lmx1a*^dr/+^*;Lmo4*^−/−^ ears (***C***, *n* = 4/4). ***D***, Anterior and posterior located cristae are present in *Lmx1a*^dr/dr^*;Lmo4*^−/−^ ears (asterisk, *n* = 4/4). It is not clear whether the two patches of crista tissues in the anterior region represent a small anterior and lateral crista or two halves of the anterior crista. ***E***–***J***, Cryosections of the crista probed for *Sox2* (***E***–***G***) and cochlea probed for *Myosin15* (***H***–***J***) transcripts in *Lmx1a*^dr/+^*;Lmo4*^+/−^ (***E***, ***H***), *Lmx1a*^+/+^*;Lmo4*^−/−^ (***F***, ***I***), and *Lmx1a*^dr/+^*;Lmo4*^−/−^ (***G***, ***J***) sections. M, Medial. For other abbreviations, please refer to Figures 2 and 3.

We further investigated the presence of sensory organs in the *Lmx1a* and *Lmo4* compound mutants because their paint-filled ears do not always reveal the presence of sensory organs. We conducted immunostaining of dissected ears or *in situ* hybridization of frozen ear sections. Although *Lmo4*^−/−^ ears did not show a bona fide ampulla in paint fill (*n* = 15/17; Table 1) or the presence of a crista in whole mounts (Fig. 5*B*, *n* = 3/3), some crista tissues were detected by *in situ* hybridization based on its location next to the utricular macula (Fig. 5*F*, asterisk, *n* = 5/6).

In contrast, an anterior-positioned crista was found in all *Lmx1a*^dr/+^*;Lmo4*^−/−^ mutants analyzed by whole mounts or sections (Fig. 5*C*, asterisk, *n* = 4/4; Fig. 5*G*, *n* = 7/7). In *Lmx1a*^dr/dr^*;Lmo4*^−/−^ double mutants, a posterior crista and two patches of crista tissues in the anterior region were evident (Fig. 5*D*, asterisks, *n* = 4/4). These results indicate that removal of the *Lmx1a* allele rescues the loss of crista phenotypes observed in *Lmo4*^−/−^ mutants in a gene-dosage-dependent manner.

### Negative regulation of *Lmx1a* by *Lmo4* restricts organ of Corti formation

In addition to the lack of cristae, a duplicated organ of Corti in the lateral wall of the shortened cochlear duct was reported in *Lmo4*^−/−^ ears (Deng et al., 2014). We investigated whether this duplicated organ of Corti phenotype also resulted from a lack of Lmo4 in regulating Lmx1a function. Two of the three *Lmx1a*^+/+^*;Lmo4*^−/−^ ears that contained a cochlea showed a relatively normal organ of Corti and no ectopic sensory tissues after whole-mount staining with anti-myosin VIIa (Fig. 5*B*). Because ectopic sensory organs in the lateral wall could be easily destroyed during whole-mount dissection, we conducted additional *in situ* hybridization analyses. Our results showed that, although we did not recover a cochlea from two of the *Lmx1a*^+/+^*;Lmo4*^−/−^ specimens, ectopic sensory tissues were usually found in three of four specimens that contained a cochlea (Fig. 5*I*, asterisk) compared with controls (Fig. 5*H*). In contrast, most of the *Lmx1a*^dr/+^*;Lmo4*^−/−^ cochleae appeared normal (Fig. 5*C*, *n* = 4/4; 5J, *n* = 7/9). In the double homozygous mutants of *Lmx1a* and *Lmo4*, in which the inner ear resembles that of the *dreher*, only one of four specimens showed a relative normal organ of Corti (Fig. 5*D*). The other three cochleae were similar to the *dreher* and *Lmx1a* conditional mutants, showing a disorganized organ of Corti and vestibular-like hair cells in the basal region. These results suggest that, although removal of a *Lmx1a* allele rescues the cochlear duct and ectopic organ of Corti formation in *Lmo4*^−/−^ ears, normal basal cochlear development is a function of Lmx1a that is independent of Lmo4.

### Overlapping expression of *Lmx1a* and *Lmo4* in the developing cristae and cochlea

**Figure 6.**
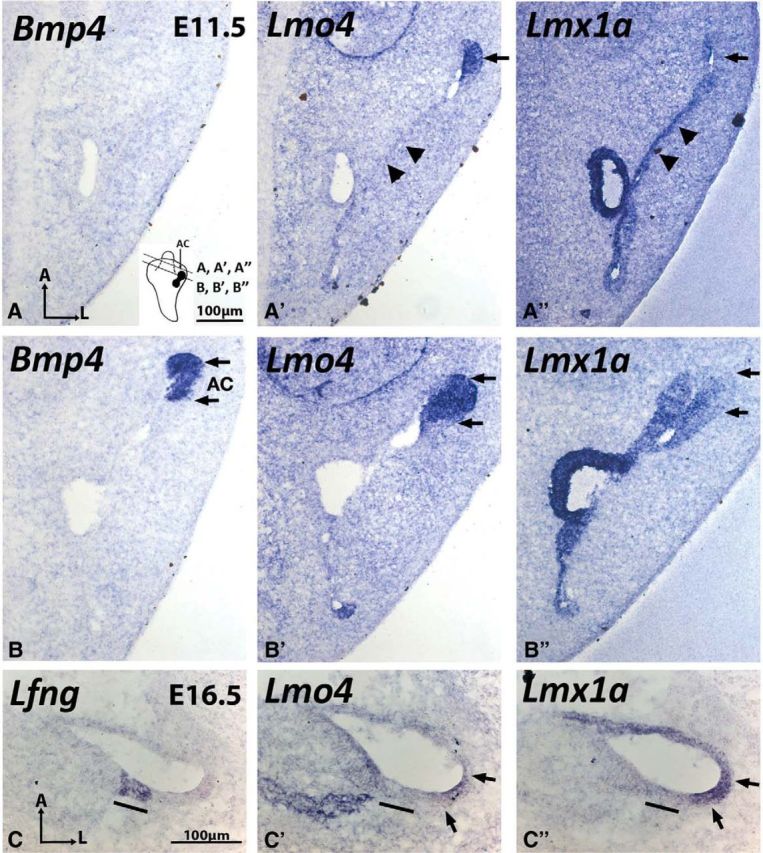
Overlapping expression domains of *Lmx1a* and *Lmo4* in the developing crista and cochlea. ***A***–***A″*** and ***B***–***B″***, are adjacent sections of the inner ear at E11.5 taken at the level of vertical canal pouch (***A***–***A″***) and presumptive anterior crista (***B***–***B″***, AC). In the *Bmp4*-negative, vertical canal pouch (***A***), *Lmo4* is expressed strongly in the rim (***A′***, arrow) but weak in the center (***A′***, arrowheads) of the canal pouch. In contrast, *Lmx1a* is strongly expressed in the center (***A″***, arrowheads) but weaker at the rim (arrow) of the canal pouch. In the *Bmp4*-positive anterior crista (***B***, double arrows), *Lmo4* (***B′***) expression is strong and *Lmx1a* (***B″***) expression is weaker. ***C***–***C″***, Expression pattern of *Lmo4* and *Lmx1a* overlap in the lateral cochlear duct at E16.5 (***C′***, ***C″***, arrows). *Lunatic Fringe* (*Lfng*) labels the organ of Corti (***C***, black bar). For orientations, please refer to Figure 2.

Although our genetic results support a plausible interaction between Lmx1a and Lmo4 in inner ear formation, we investigated whether this hypothesis is supported by an overlap of expression patterns in the prospective structures postulated to be dependent on Lmx1a and Lmo4 interactions. In the canal pouch, which develops into anterior and posterior canals, *Lmx1a* was primarily expressed in the center of the canal pouch, where most of the tissues are destined for resorption (Fig. 6*A′′*, arrowheads). In contrast, *Lmo4* hybridization signals were primarily detected at the rim of the pouch, which forms the canals (Fig. 6*A′*, arrow), and signals were only weakly detected in the center of the canal pouch (Fig. 6*A′*, arrowheads). In the *Bmp4*-positive presumptive anterior crista at E11.5, *Lmx1a* and *Lmo4* were coexpressed, although the level of *Lmx1a* expression seems lower compared with that of *Lmo4* (Fig. 6*B–B′′*, arrows). These expression results are consistent with the hypothesis that Lmx1a and Lmo4 interact to form the cristae and canals. Whereas it is not clear when the ectopic sensory organ in the organ of Corti is specified in the *Lmo4* knock-out mutants, we observed overlapping expression between *Lmo4* and *Lmx1a* in the lateral wall of the cochlear duct at E16.5 (Fig. 6*C–C′′*), which is consistent with the location of ectopic sensory patches found in *Lmo4* knock-outs (Deng et al., 2014).

### Ectopic *Lmx1b* expression in chicken inner ears affects crista development

**Figure 7.**
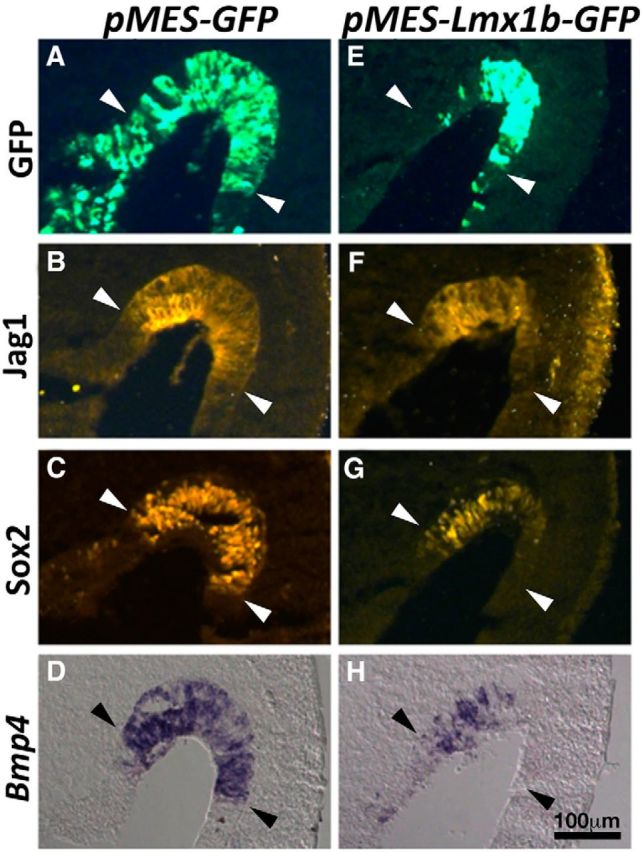
Ectopic *Lmx1b* expression in the presumptive anterior crista of the chicken inner ear downregulates crista markers *Bmp4*, Jag1, and Sox2. Sections of chicken inner ear electroporated with *pMES-GFP* (***A***–***D***) or *pMES-Lmx1b-GFP* (***E***–***H***) plasmids at E3.5 and were harvested 24 h later and processed for anti-Jag1 (***B***, ***F***) and anti-Sox2 (***C***, ***G***) immunostaining and *Bmp4* gene expression (***D***, ***H***). The levels and domains of Jag1 and Sox2 immunoreactivities and *Bmp4* hybridization signals in the prospective anterior crista (marked by arrowheads) are reduced in specimens electroporated with *pMES-Lmx1b-GFP* (***E***–***H***) compared with *pMES-GFP* controls (***A***–***D***).

Based on the hypothesis that Lmx1a is required to be downregulated in order for crista to form properly, excess Lmx1a should be detrimental to crista formation, as predicted in the case of *Lmo4* knock-outs. To test this hypothesis further, we electroporated an expression vector encoding *Lmx1b* into the developing chicken anterior crista *in ovo* because *Lmx1b* is the predominant *Lmx1* member expressed in the chicken inner ear (Giráldez, 1998; Abelló et al., 2010). The plasmids *pMES-Lmx1b-GFP* or *pMES-GFP* were electroporated into the presumptive anterior crista region of the right otocyst at E3.5 and embryos were harvested 24 h later. Samples with good targeting to the sensory region and high GFP expression were collected and analyzed (Fig. 7*A*,*E*). The expression of *Bmp4*, Jag1 and Sox2 were compared in adjacent cryosections. Hybridization signals of *Bmp4* were decreased with *pMES-Lmx1b-GFP* plasmid (Fig. 7*H*, *n* = 16/19). Anti-Jag1 immunostaining was decreased in 81% (Fig. 7*F*, *n* = 13/16) and anti-Sox2 staining was only decreased in 50% of the samples analyzed (Fig. 7*G*, *n* = 8/16). In contrast, expression patterns of most of the specimens electroporated with the control plasmids, *pMES-GFP*, were not changed (Fig. 7*B–D*, *Bmp4*, *n* = 17/18; anti-Jag1, *n* = 18/18; anti-Sox2, *n* = 15/17). These Lmx1b gain-of-function results support the hypothesis that Lmx1 functions are normally required to be downregulated during crista development.

## Discussion

### Inner ear source of *Lmx1a* is important for inner ear patterning

During embryogenesis, the hindbrain develops earlier than the ear. The representation of mutant mouse strains with both hindbrain and inner ear defects has prompted the proposal that hindbrain influences ear formation (Deol, 1966). Over the years, it has been shown that the hindbrain provides multiple signals such as Mafb, Fgf3, and Wnts, which mediate inner ear development (Riccomagno et al., 2005; Choo et al., 2006; Hatch et al., 2007). Although *dreher* is among one of the early mouse mutants described to have both hindbrain and inner ear defects, our tissue-specific knock-out studies suggest that the inner ear defects in the *dreher* mutants are primarily caused by the loss of *Lmx1a* in the inner ear rather than roof plate defects in the hindbrain. Furthermore, the regional reciprocal negative regulation of Lmx1a and Lmo4 described here also supports a role of Lmx1a within the inner ear because Lmo4 has been demonstrated to function within the inner ear (Deng et al., 2010). Nevertheless, due to the minor hindbrain phenotypes observed in the two conditional mutants, we cannot rule out a contribution of the hindbrain source of Lmx1a to inner ear formation.

### Lmo4 negatively regulates Lmx1a in mediating crista formation

**Figure 8.**
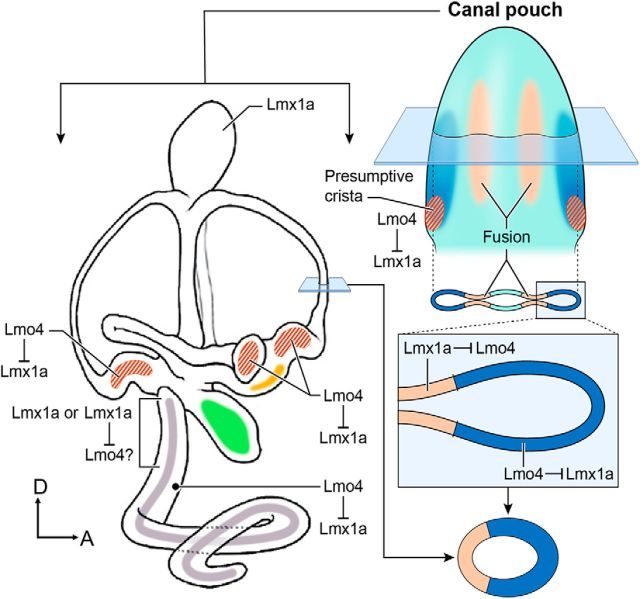
Summary diagram of the requirements of Lmx1a and Lmo4 in inner ear formation. The vertical canal pouch (right) gives rise to the anterior and posterior canals. The epithelial cells in the center region of each prospective canal (tan color) fuse and resorb resulting in the rim of the canal pouch (blue color) forming the anterior and posterior canals. Lmo4 negatively regulates Lmx1a to form the three cristae (red stripes), anterior canal, and proper shape of the utricle (yellow color) and to inhibit ectopic sensory tissue formation in the cochlear duct. In contrast, Lmx1a negatively regulates Lmo4 to mediate resorption in the vertical canal pouch. The endolymphatic duct formation requires Lmx1a independent of Lmo4, whereas the hair cells in the basal cochlear region may require Lmx1a independently or its negative regulation of Lmo4. Organ of Corti is shown in gray and the saccule in green. For orientations, please refer to Figure 2.

Our phenotypic analyses of the single and compound mutants of *Lmx1a* and *Lmo4* indicate that these two genes interact genetically to mediate formation of various inner ear structures. In addition, our results suggest that Lmx1a has functions in the inner ear that are independent of Lmo4 (Fig. 8).

Several lines of evidence indicate that Lmo4 negatively regulates Lmx1a in crista formation (Fig. 8, red stripes). Cristae fail to develop in *Lmo4* knock-out ears and this phenotype is rescued by the removal of *Lmx1a* in a dose-dependent manner: recovery of more crista tissues with the absence of both alleles of *Lmx1a* than one (Fig. 5*C*, *D*). Consistently, loss of Lmx1a function did not affect formation of the cristae. These results, together with the observation of high levels of *Lmo4* but low levels of *Lmx1a* transcripts detected in normal presumptive cristae, suggest that Lmx1a's functions need to be downregulated in the presumptive cristae in order for each crista to form properly. This hypothesis is further supported by the demonstration that ectopic *Lmx1b* downregulated crista-specific genes in the chicken inner ear (Fig. 7).

### Multiple roles of Lmx1a and Lmo4 in canal formation

In contrast to the presumptive cristae, the requirement of Lmx1a and Lmo4 in canal formation is more complicated and appears to be mutually inhibitory. First, the high frequency of the anterior canal recovery in *Lmo4* knock-out ears with the absence of one copy of *Lmx1a* suggests that Lmo4 negatively regulates Lmx1a in the anterior canal formation, analogous to crista formation. However, the recovery of canals in *Lmx1a*^dr/dr^*;Lmo4*^+/−^ compared with *Lmx1a*^dr/dr^ ears suggests that Lmx1a also negatively regulates Lmo4 in canal formation. These results suggest that the levels of Lmx1a and Lmo4 are tightly titrated during canal formation (Fig. 8, right).

Where could the titration of Lmx1a and Lmo4 levels be taking place during canal formation? The three semicircular canals form from two epithelial out-pockets during the otocyst stage known as the canal pouches. The vertical canal pouch gives rise to the anterior and posterior canal and the lateral pouch gives rise to the lateral canal. Within the canal pouch, the two-opposing epithelia in the center of each prospective canal resorb (Fig. 8, tan color) and leave behind the rim of the canal pouch to form the toroidal-shaped canal (Fig. 8, blue color) (for review see, Wu and Kelley, 2012). In the case of Lmx1a and Lmo4, we propose that the strong *Lmo4* expression at the rim of the canal pouch is required to inhibit Lmx1a function to form the canals. By removing one allele of *Lmx1a*, the loss of canal phenotype in *Lmo4* knock-outs is alleviated, resulting in the recovery of the anterior canal (Fig. 4*D*,*E*). In contrast, we propose that the strong *Lmx1a* expression in the center of the canal pouch is required to mediate the resorption process and to block the function of Lmo4. Although the nonresorption phenotype in *Lmx1a* mutants is also alleviated by the removal of one allele of *Lmo4*, the extent of rescue is variable and not even consistent between two ears of the same embryo (Fig. 4*H*). These results suggest that the negative regulation of Lmx1a by Lmo4 could be indirect and may involve other cofactors or downstream pathways. In addition, the inhibition can occur between the resorption and the prospective canal domains because other studies have shown that these domains reciprocally inhibit each other (Abraira et al., 2008).

The presence of the canal pouch in the *Lmx1a* and *Lmo4* double-null mutants indicates that neither Lmx1a and Lmo4 is required to establish the canal pouch formation, but they function to pattern the canals. Furthermore, the similarity in phenotypes between *Lmx1a* mutants and *Lmx1a* and *Lmo4* double mutants suggests that *Lmx1a* functions are epistatic to *Lmo4*.

### Multiple roles of *Lmo4* and *Lmx1a* in regulating organ of Corti formation in the cochlea

Similar to the vestibular system, Lmo4 and Lmx1a interact to form the cochlea. The lack of *Lmo4* causes an ectopic organ of Corti in the lateral wall of the cochlear duct (Deng et al., 2014). Lmo4 is thought to function upstream of most known genes involved in the organ of Corti patterning such as *Sox2*, *Jag1*, and *Notch1* (Deng et al., 2014). A number of mechanisms have been proposed for Lmo4's function in the lateral cochlea, including downregulation of *Bmp4* expression, negative regulation of LIM-TFs and positive regulation of multimeric transcriptional complexes by bridging with factors such as GATA and basic helix-loop-helix proteins (Deng et al., 2014). Here, we provide genetic evidence that *Lmo4* mediates its function in the lateral cochlea by negatively regulating *Lmx1a*. Considering that the length of the cochlear duct and the ectopic organ of Corti phenotype are rescued with the absence of one allele of *Lmx1a* in the *Lmo4* knock-out mutants suggest that a major role of Lmo4 in the cochlear duct is to restrict ectopic sensory tissue formation in the cochlea by negatively regulating Lmx1a. In support of the genetic evidence, *Lmx1a* and *Lmo4* expression domains overlap in the lateral cochlea where ectopic sensory patches are located.

Although we show that Lmo4 negatively regulates Lmx1a functions in restricting sensory fate in the lateral cochlea, the conversion to vestibular-like hair cells in the basal cochlear region of the *Lmx1a* mutants appears to be only partially rescued in the *Lmx1a* and *Lmo4* double mutants (*n* = 1/4). And, whereas the sample size is small, these results raise the possibility that excess Lmo4 in *Lmx1a* mutants may cause the basal cochlear phenotype in *Lmx1a* mutants and that this phenotype is alleviated partially in *Lmx1a* and *Lmo4* double-null cochlea. Alternatively, Lmx1a's function in the basal cochlear region is independent of Lmo4, similar to its role in establishing the endolymphatic duct (Fig. 8).

In summary, it has been well established that the combination of LIM-TFs and their cofactors determine the specificity of transcriptional targets, which can lead to cells adopting different fates. The presence of LIM-TFs such as Islet1 or combination of Islet1 and Lhx3 dictates the type of motor neurons that forms in the spinal cord, whereas the presence of Lhx3 alone is important for forming the V2 interneurons. Lmo4 is involved in the regulation of these fates by titrating the levels of Islet1 in motor neuron formation and mediating formation of V2 interneuron subtypes (Gill, 2003; Lee et al., 2008; Joshi et al., 2009; Song et al., 2009). Although the inner ear also expresses transcription factors such as Islet1 and Gata3 that are known to interact with Ldb proteins in other systems, it is not clear whether they interact with Lmx1a and/or Lmo4. Nevertheless, our results have robustly identified an important piece of the puzzle: Lmx1a and Lmo4 interact to regulate one another negatively in the formation of the inner ear.

#### 

##### Note added in proof.

Since this manuscript was in review, a paper on Lmx1a's role in shaping the sensory organs of the inner ear was published by Mann et al. doi: https://doi.org/10.7554/elife.33323.
